# RNA sequencing analysis of the developing chicken retina

**DOI:** 10.1038/sdata.2016.117

**Published:** 2016-12-20

**Authors:** Christophe J. Langouet-Astrie, Annamarie L. Meinsen, Emily R. Grunwald, Stephen D. Turner, Raymond A. Enke

**Affiliations:** 1 Department of Biology, James Madison University, Harrisonburg, Virginia 22807, USA; 2 Department of Public Health Sciences University of Virginia, Charlottesville, Virginia 22908, USA; 3 Center for Genome & Metagenome Studies, James Madison University, Harrisonburg, Virginia 22807, USA

**Keywords:** Transcriptomics, Differentiation, Data publication and archiving, Computational neuroscience

## Abstract

RNA sequencing transcriptome analysis using massively parallel next generation sequencing technology provides the capability to understand global changes in gene expression throughout a range of tissue samples. Development of the vertebrate retina requires complex temporal orchestration of transcriptional activation and repression. The chicken embryo (*Gallus gallus*) is a classic model system for studying developmental biology and retinogenesis. Existing retinal transcriptome projects have been critical to the vision research community for studying aspects of murine and human retinogenesis, however, there are currently no publicly available data sets describing the developing chicken retinal transcriptome. Here we used Illumina RNA sequencing (RNA-seq) analysis to characterize the mRNA transcriptome of the developing chicken retina in an effort to identify genes critical for retinal development in this important model organism. These data will be valuable to the vision research community for characterizing global changes in gene expression between ocular tissues and critical developmental time points during retinogenesis in the chicken retina.

## Background & Summary

Next-generation sequencing technology has allowed for extensive transcriptome analysis of a wide range of species both with and without reference genome assemblies^1,2^. These RNA sequencing (RNA-seq) analyses have become the gold standard for in depth characterization of global changes in gene expression as well as the accumulation of novel transcript isoforms. Once made publicly available, genome-wide experiments also provide the research community with valuable data that can be subsequently mined to further scientific knowledge. Detailed curation of these datasets is paramount for accurate interpretation, widespread dissemination, and repurposing of data.

The experiment described here is part of a larger ongoing project within the James Madison University’s Center for Genome & Metagenome Studies (CGEMS) investigating transcriptional regulation in the developing vertebrate retina. Within the developing retina, precise transcriptional regulation is critical for proper differentiation of specialized subclasses of retinal neurons and glial support cells^3^. Insights into these complex regulatory networks are critical for further understanding molecular mechanisms that drive human retinal development as well as for crafting novel strategies to combat blinding human diseases that affect the retina. Because developmental processes are highly conserved among vertebrate species, the chicken (*Gallus gallus*) embryo is a reliable and practical model system for studying organogenesis. Developmental staging of the chicken embryo has been characterized in meticulous detail for 65 years^4^. Embryo development is rapid, completing its entire program from blastula to hatchling in 21 days. Chick embryos are particularly useful for studying ocular development compared to other model organisms as their developmental eyes are easily accessible for experimentation due to their relatively large size. Recent resequencing and improvement of the chicken genome assembly combined with newly developed molecular tools for genetic manipulation of this model system have contributed to a renaissance of using the chicken embryo as a robust model to study retinal development^5^.

**Figure 1:**
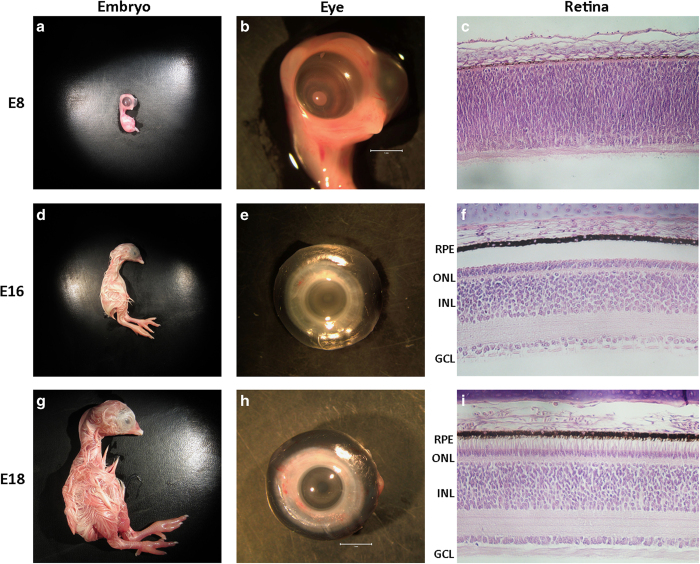
Overview of Gallus gallus embryo, eye, and retinal development. Image of embryonic day 8 (E8), E16 and E18 embryos (**a**,**d**,**g**), eyes (**b**,**e**,**h**) and H+E stained retinal cross sections (**c**,**f**,**i**). Cross section abbreviations: Retinal Pigmented Epithelium (RPE), Outer Nuclear Layer (ONL), Inner Nuclear Layer (INL), and Ganglion Cell Layer (GCL).

During chick retinal development, embryonic day 8 (E8) and embryonic day 18 represent early and late retinal developmental time points with respect to cellular differentiation of retinal neurons. The early E8 retina is packed with multipotent precursor cells while the E18 retina is nearly mature with photoreceptor (PR), bipolar, amacrine, horizontal, and ganglion cell neurons as well as Muller glial cells having differentiated from these multipotent precursors (Fig. 1) (ref. 5). Precursor cells yet to exit the cell cycle as well as each of these specialized retinal cell types are known to express developmental and cell type-specific genes, however the full detail of these specific expression patterns is yet to be defined^6^. Only recently have transcriptome experiments began to be employed as a tool to dissect global changes in gene expression during chick retinal development. A recent study using an elegant reporter system followed by cell capture and RNA-seq analysis, characterized differential gene expression of rod and cone photoreceptor differentiation during chick retinal development^7^. Focusing solely on this one important class of retinal neuron, Enright and colleagues were able to identify hundreds of differentially expressed genes involved in diverse cellular processes contributing to the birth of chicken photoreceptors^7^. Currently, there are no publicly available data sets characterizing the whole chicken retina transcriptome during development.

**Figure 2:**
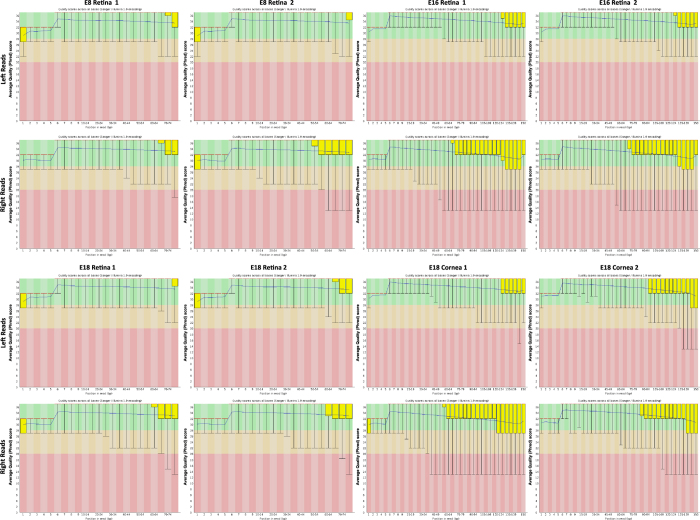
Quality assessment of raw FASTQ sequence data for 150 bp paired end left (1) and right (2) reads. Box and whisker plots demonstrate the distribution of per base quality for each left and right read position read for each of the 16 samples. Mean value is indicated by the blue line and the yellow box represents the interquartile range (25–75%) with the lower and upper whiskers represent the 10 and 90% points respectively. Plots were generated using the FastQC program (see Code Availability 1).

**Figure 3:**
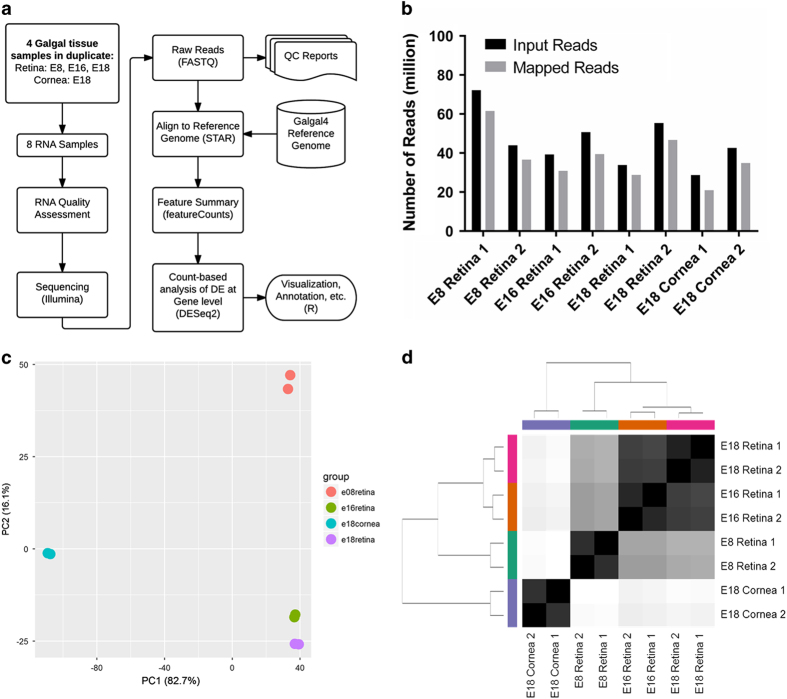
Experimental overview and assessment of read mapping and sample variance. (**a**) Flowchart of the RNA-seq experiment and data analysis. (**b**) STAR Alignment total number of raw reads compared to number of mapped reads listed per sample. Additional details about the alignment are listed in Table 2. (**c**) Principal Component Analysis (PCA) Biplot of RNA samples. The first principal component (PC1) is the tissue type (cornea versus retina) and the second principal component (PC2) is the embryonic stage day. (**d**) Hierarchical clustering analyses performed using DESeq2 rlog-normalized RNA-seq data^9,17^. Color code refers to the distance metric used for clustering with white being the lowest correlation value and black being the largest correlation value.

This focus of this project is to characterize the mRNA transcriptome of the developing chicken retina in an effort to identify genes critical for differentiation of the six major retinal cell types as well as their derivative cell subtypes in this important model organism. The developmental points chosen were E8 (Fig. 1a–c), E16 (Fig. 1d–f), and E18 (Fig. 1g–j), which provides transcriptional information for early, middle/late, and late retinal development respectively. E18 whole cornea (Fig. 1h) was also included in this analysis as a non-retinal reference tissue. These analyses were conducted using Illumina RNA-seq in tandem with a bioinformatics pipeline to ensure sequence quality (Fig. 2) and for robust eukaryotic transcriptome analysis (Fig. 3).

## Methods

### Embryos

All embryo experiments were conducted with the approval of the James Madison University Institutional Animal Care and Use Committee and in accordance with the National Institutes of Health guide for the care and use of laboratory animals. Fertilized pathogen free commercial Cobb/Hubbard F1 hybrid eggs were obtained from George’s Hatchery (Harrisonburg, VA) and incubated in a rocking chamber held at 38 °C and 50–60% humidity until specified incubation days.

### Tissue processing, histology & imaging

Chicken embryos were harvested and euthanized at specified days incubated by decapitation. Intact eyes were enucleated and placed in cold PBS. Whole embryos were imaged using an iPhone6 and whole eyes were imaged using a Stemi SV 6 stereo microscope (Zeiss) equipped with 18.2 Color Mosaic camera (SPOT). Eyecups were prepared by piercing and cutting around the limbus to dissect away the anterior segment, lens, and vitreous. Isolated corneas were saved for subsequent RNA extraction. For histology, eyecups were fixed in 4% paraformaldahyde in 1× PBS for 25 min on ice and then equilibrated for 2–24 hours in 25% sucrose in 1× PBS. Equilibrated eyecups were transferred into a 2:1 mixture of 25% sucrose:OCT compound (Electron Microscopy Sciences) on ice for 30 min and then flash frozen in the same solution in a Tissue-Tek Cryomold (Sakura Finetek) and stored at −80 °C. 10 μm thick frozen serial sections were prepared using a CM3050 S Research Cryostat (Leica) with the object and chamber temperatures set to −22 °C and −28 °C respectively. Frozen sections were thawed, H&E stained, and imaged using an EclipseTE2000 inverted microscope (Nikon) and processed with NIS Elements software (Nikon). For retinal dissection, eyecups were incubated for 20 min in HBSS modified media without calcium or magnesium (HBSS -Ca,-Mg;HyClone) at 37 °C to dissociate the retinal pigment epithelium (RPE) layer from the outermost layer of the retina. Retinas were then isolated by tearing away the sclera and gently peeling away the RPE layer. Isolated retinas and corneas were briefly rinsed in cold HBSS -Ca, -Mg. Retinas were immediately transferred to RLT+ lysis buffer (Qiagen; AllPrep kit) containing 2-Mercaptoethanol (Sigma) and vortexed vigorously to dissociate the tissue. Corneas were flash frozen and ground into a fine powder using a mortar and pestle prior to being transferred to RLT+/BME lysis buffer solution and vortexed. Samples were stored long term in lysis buffer at −80 °C.

### Total RNA isolation

Total RNA was collected from eight embryonic chicken ocular tissues (Table 1). Whole retinas were harvested from E8 (Fig. 1a–c), E16 (Fig. 1d–f), and E18 (Fig. 1g–j) developing chicken embryos as well as whole corneas collected from E18 embryos (Fig. 1h). Duplicates were obtained for each time point and total RNA was extracted from samples using a Qiagen AllPrep Mini Kit with an on column DNaseI treatment step per the manufacturer’s instructions. Isolated RNAs were eluted in nuclease free water, validated for quality and quantity using UV spectrophotometry, and stored long term at −80 °C. RNAs with a OD260/280 ratio between 1.9 and 2.1 were deemed high quality.

### RNA preparation and sequencing

Total RNA samples chosen for characterization of global mRNA expression were submitted to the Cold Spring Harbor Laboratory DNA Sequencing Center for Bioanalyzer quality control analysis (Agilent) and Illumina Next Generation Sequencing. All submitted samples had RNA integrity number (RIN) >8. Stranded TrueSeq libraries with poly dT enrichment were prepared from total RNA from each of the four samples in biological duplicate according to the manufacture’s protocol. The resulting average size of the cDNA libraries was approximately 300 bp. Libraries for the 8 cDNA samples were sequenced using the Illumina NextSeq 500 sequencing platform yielding 28.6–72.2 million 125 bp paired end sequence reads per sample (Table 2; Fig. 3b).

### Quality validation and read mapping

Between 28.7 and 72.2 million paired end sequence reads were obtained per sample from the Cold Spring Harbor Laboratory DNA Sequencing Center (Table 2). Quality of individual sequences were evaluated using FastQC analysis (see Code Availability 1), including per base sequence quality analysis which plots the Phred quality score distribution on the y axis for each read generated per sample for each nucleotide base call plotted on the x axis (Fig. 2). Figure 2 demonstrates that all 16 FASTQ sequencing files have an average per base Phred score >28, a conventional threshold denoting high quality base calls. Figure 3a demonstrates our experimental overview including the bioinformatics pipeline employed following quality validation of sequence reads. High quality sequence reads were aligned to the UCSC Gallus gallus reference genome (galGal4) using STAR^1^ for ultrafast transcript assembly (see Code Availability 2). The percentage of uniquely mapped reads ranged from 73 to 85% (Table 2; Fig. 3b). Further quality validation of the data set was assessed using mapped reads from each sample.

### Data transformation and downstream analysis

Differential gene expression between samples was quantified at the gene level using the read summarization program featureCounts (see Code Availability 3)^2^. From this point on, all data analysis was conducted using R programing language and related packages. The output matrix from featureCounts^8^ was input into the bioconductor package DESeq2 (see Code Availability 4)^9^. This package was used to normalize the count data with a negative binomial distribution and values were log_2_ transformed. Statistical plots principal component analysis (PCA) and distance matrix analysis were generated with the same package to assess variance between sample groups and sample replicates (Fig. 3c,d). Ensembl gene IDs mapping to predicted genes were excluded and the Benjamini-Hochberg False Discovery Rate^10^ procedure was used to re-estimate the adjusted *P*-values for Ensembl gene IDs^11^ mapping to known genes.

### Code availability

The following software and versions were used for quality control and data analysis as described in the main text:

1. FastQC, version 0.11.4 was used for quality analysis of raw FASTQ sequencing data: http://www.bioinformatics.babraham.ac.uk/projects/fastqc/
2. STAR, version 2.5.1b was used for mapping of sequence reads to the chicken galGal4 genome assembly: https://github.com/alexdobin/STAR/releases
3. FeatureCounts, version 1.5.0 was used for differential gene expression analysis: http://bioinf.wehi.edu.au/featureCounts/
4. DESeq2, version 1.6.2 was used for normalization and visualization of differential gene expression analysis output: http://bioconductor.org/packages/DESeq2

All code used for quality assessment and data analysis in this study is available at: https://github.com/stephenturner/langouetastrie-scidata2016-chicken-rnaseq-retina-code.

## Data Records

Raw FASTQ files for the RNA-seq libraries were deposited to the NCBI Sequence Read Archive (SRA) (Data Citation 1), and have been assigned BioProject accession PRJNA275440. Output from the transcripts were deposited to the NCBI Gene Expression Omnibus (GEO) with accession number GSE65938 providing access to all the data files (Table 1; Data Citation 2). Supplementary file GSE65938_GEOENKE-RNASEQ-normcounts.csv.gz contains the genome, counts, and normalized counts used to generate the statistical plots (Data Citation 2).

## Technical Validation

### Quality control-RNA integrity

Quality of total RNA fractions was assessed using an Agilent Bioanalyzer to calculate a RNA Integrity Number (RIN). The RIN algorithm determines the RNA quality of the samples with the highest quality having a score of 10. Conventional to NGS analysis, only RNA samples with a RIN >8 were used for sequencing analysis.

### RNA-Seq raw data quality

FastQC per base sequence quality analysis demonstrates mean Phred quality scores are well within the acceptable range for downstream analysis (Fig. 2). Between 21.0–61.5 million reads mapped reads were mapped to the chicken reference galGal4 genome assembly (Table 2). PCA biplot and distance matrix confirm the similarity between biological replicates and variability between developmental time points respectively. In addition, the PCA plot displayed that sample type (PC1) and embryonic developmental day (PC2) account for 98.8% of the variability in gene expression (Fig. 3c,d).

## Usage Notes

The bioinformatics pipeline applied to our data set outlined in Fig. 3a was achieved using a collection of freely available, open access tools. These analyses however, are interchangeable with many other currently available tools. Our raw fastq data can be aligned to any available chicken reference genome, including the most recent 2011 galGal4 assembly, using a variety of freely available aligners. In this study we used the STAR genome aligner^12^, however, similar analysis may be achieved using the ‘new tuxedo’ pipeline^13^. Alignment of the fastq data in the form of bam files can be viewed using popular genome browser such as the UCSC Genome Browser^14^, the Ensembl Browser^11^ or the Broad Institute’s Integrative Genome Viewer (IGV)^15,16^. Here our differential gene expression analysis was carried out using DESeq2 (refs 9,^17^), however other publicly available packages such as egdeR^18^ or CuffDiff^1^ may also be used for this analysis. An alternative to using genome aligners is to employ an alignment-free transcript quantification^19,20^ step followed by gene-level summation^21^ then differential expression analysis such as DESeq2 (refs 9,^17^). An alignment-free pipeline reduces the time of analysis as well as required computing power which may be beneficial for some users^19,20^.

Our data set will be useful for a variety of studies investigating developmental and tissue-specific changes in gene expression in the vertebrate retina. There are however, several considerations that must be taken into account when using these data for downstream analysis. First, RNAs were extracted from whole retina or whole cornea without any enrichment for cell type. Therefore, resulting downstream analysis will be representative of heterogeneous mixtures of differing cell types within these tissues. Second, cDNA libraries were prepared using a poly dT primer, thus the data set is representative of only polyadenylated mRNA transcripts and does not represent non-coding RNA or other non-polyadenylated cellular transcripts. Additionally, usage of poly dT priming introduces a bias towards overrepresentation of the 3’ end of transcripts, particularly in the case of large transcripts. Finally, the quantity of sequenced and mapped reads per sample in this study (Table 2; Fig. 3b) is sufficient for robust differential gene expression analysis, however, is below the conventional threshold for thorough differential isoform analysis^22^. Taking these considerations into account, these data will be a useful resource for the vision research community to thoroughly investigate critical changes in gene expression that take place during the complex process of vertebrate retinal development. Additionally, these data will be available to explore important tissue-specific comparisons in gene expression patterns between the retina and the cornea, two clinically relevant ocular tissues.

## Additional information

**How to cite this article:** Langouet-Astrie, C. J. *et al.* RNA sequencing analysis of the developing chicken retina. *Sci. Data* 3:160117 doi: 10.1038/sdata.2016.117 (2016).

**Publisher’s note:** Springer Nature remains neutral with regard to jurisdictional claims in published maps and institutional affiliations.

## Supplementary Material



## Figures and Tables

**Table 1:** RNA-seq profiling to evaluate developmental and tissue-specific retinal gene expression.

**Subject**	**Source**	**Sample**	**Method 1**	**Method 2**	**GEO Accession#**
Chicken 1	embryonic day 8 retina	E8 retina 1	total RNA extraction	RNA-seq	GSM1611468
Chicken 2	embryonic day 8 retina	E8 retina 2	total RNA extraction	RNA-seq	GSM1611469
Chicken 1	embryonic day 16 retina	E16 retina 1	total RNA extraction	RNA-seq	GSM1611466
Chicken 2	embryonic day 16 retina	E16 retina 2	total RNA extraction	RNA-seq	GSM1611467
Chicken 1	embryonic day 18 retina	E18 retina 1	total RNA extraction	RNA-seq	GSM1611470
Chicken 2	embryonic day 18 retina	E18 retina 2	total RNA extraction	RNA-seq	GSM1611471
Chicken 1	embryonic day 18 cornea	E18 cornea 1	total RNA extraction	RNA-seq	GSM1611472
Chicken 2	embryonic day 18 cornea	E18 cornea 2	total RNA extraction	RNA-seq	GSM1611473

**Table 2:** RNA-seq read statistics.

**Sample Name**	**Sequencer**	**Read Length (bp)**	**Million read-pairs**	**Uniquely mapped reads (%)**
E8 retina 1	Illumina NextSeq 500	2×125	72.21	85.2
E8 retina 2	Illumina NextSeq 500	2×125	43.93	83.4
E16 retina 1	Illumina NextSeq 500	2×125	39.32	78.5
E16 retina 2	Illumina NextSeq 500	2×125	50.71	77.8
E18 retina 1	Illumina NextSeq 500	2×125	33.89	84.9
E18 retina 2	Illumina NextSeq 500	2×125	55.36	84.4
E18 cornea 1	Illumina NextSeq 500	2×125	28.73	73.1
E18 cornea 2	Illumina NextSeq 500	2×125	42.63	81.7
